# Rush Medical College

**Published:** 1845-03

**Authors:** J. H. Bird


ILLINOIS

MEDICAL & SURGICAL JOURNAL.

VOL. I. MARCH, 1845. NO. 12.

### RUSH MEDICAL COLLEGE.

### PROF. BRAINARD'S SURGICAL CLINIC. FEB. 3d, 1845.

[Reported for the Journal, by J. H. Bird.]

Case 1.—Mrs. M——, having an obstruction of the lachrymal duct presented herself for relief.

Dr. B. remarked, that the lachrymal passage was formed by the lachrymal of each lid, the lachrymal sac and the nasal duct. The lachrymal canals, commencing at the puncta lachrymalia by capillary openings, continue their course inwards and downwards to empty into the lachrymal sac, which is of an oblong shape, and situated in the groove made by the os unguis and the nasal process of the superior maxillary bone, behind the tendon of the orbicularis muscle, having an inclination downwards, backwards and inwards; this sac connects with the nasal duct, which is formed by the inner wall of the maxillary sinus, os unguis, and inferior turbinated bone. The whole canal is lined by a delicate mucous membrane, besides which the sac has a fibrous membrane. The obstruction to the flow of tears, may be in the lachrymal canals, sac, or nasal ducts; may be congenital, or the result of inflammation, &c. As I have sufficiently enlarged upon the subject in my lectures, I will confine myself to the case before us. The effect of inflammation of the mucous membranes, you are aware, is to produce thickening and induration. In the present case it is the result of inflammation. The symptoms of the obstruction are, a discharge of the tears over the cheek, dryness of the corresponding nostril, and fullness at the inner angle of the eye; if the obstruction be not removed, there is at length ulceration of the sac and a fistulous opening formed, of the effects of which I have already spoken. In the early stages of the obstruction our treatment is quite successful, in later stages an operation is necessary. In cases of inflammation, the usual local and general remedies are necessary, and the inhalation of the following as a snuff, is very efficacious, viz: ℞.—Hellebore alb., grs. jv; hydrg. chlorid., ʒj; sach. alb., ʒjv—M.

In cases of obstruction from syphilis, &c., the cause must first, as far as possible, be removed. In chronic cases an operation is required. It consists in laying open the sac at the inferior margin of the tarsal ligament, by a bistoury, and introducing some substance to dilate the passage.

Dupuytren introduced small gold tubes into the passage, and many surgeons have followed his example, but it was not attended with great success, and is somewhat dangerous. I saw, while at Paris, several of these tubes extracted from situations somewhat removed from the nasal duct. Small pieces of catgut and elm-bark have been used, but with no greater success. I prefer curved silver stylets with small heads which retain them in their situations; these answer all the indications, and can be removed at any period. But gentlemen, the result is not as favorable as is to be desired.

Our patient has submitted to the operation twice before, but with relief for limited periods.

The Dr. then performed the operation and the patient was dismissed.

---

Case 2.—Miss ——, ætat 8 years, with chronic enlargement of the tonsils. Dr. B. remarked, I suppose you have been told of the effects of chronic enlargement of the tonsils by my colleagues; so it will not be necessary for me to speak of any thing but the operation. It is an operation which although not painful, is attended with some inconvenience from the situation of the large vessels, &c., and the peculiar states of the glands where hæmorrhage might occur. Although not connected with the subject, I cannot refrain from speaking of some of the means to be resorted to, in cases of hemorrhage from wounds, &c., of the tonsils.

Several years since, I removed the right tonsil from a lady. Some hours afterwards, I was called in haste to see her. I found an alarming hemorrhage from the tonsil. After resorting to the regular prescribed remedies to no purpose, I feared the necessity of a ligature of the carotid, when it occurred to me to make compression upon the part. Introducing the fingers of one hand into the mouth, and the other hand corresponding externally, I after a compression of half an hour, succeeded in entirely arresting the hemorrhage.

There are many instruments in use for extraction of the tonsils; one called the "guillotine" is the best for children, but I prefer the forceps and scissors for adults. The Dr. then performed the extraction, and the patient was dismissed.

---

Case 3.—Miss ———, with converging strabismus of right eye.

Remarks. Gentlemen, this is my forty-third case, and, as I have before remarked, I am well satisfied of the value of the operation. As this case is quite similar to others that have been before you, I will not detain you by further remarks. The operation was then performed, and the eye resumed its normal position.

